# Validity of a Combined General and Oral Health Indicator for Vulnerability

**DOI:** 10.1016/j.identj.2026.109729

**Published:** 2026-07-02

**Authors:** Han-Nah Kim, Shiho Kino, Nam-Hee Kim

**Affiliations:** a Department of Dental Hygiene, College of Health Science, Kangwon National University, Samcheok, Republic of Korea; b Department of Dental Hygiene, Mirae Campus, Yonsei University, Wonju, Republic of Korea; c Department of Preventive Oral Health Care Sciences, Graduate School of Medical and Dental Sciences, Institute of Science Tokyo, Tokyo, Japan

**Keywords:** Self-rated oral health, Self-rated health, Chewing difficulty, Health inequalities, Community-level determinants, Multidimensional vulnerability

## Abstract

**Introduction and aims:**

Self-rated health (SRH) is widely used for population health monitoring but may miss oral health–related vulnerability. We examined whether combining SRH with self-rated oral health (SROH) improves identification of multidimensional vulnerability among adults aged ≥45 years.

**Methods:**

We used 2024 Korea Community Health Survey data to create a four-category SRH–SROH indicator: good both, poor oral only, poor general only, and poor both. Survey-weighted multinomial logistic regression assessed associations with sociodemographic, behavioural, health, oral healthcare access, and community-level factors. Chewing difficulty was excluded from regression models and used as an external functional validation outcome. Incremental validity was assessed by comparing Nagelkerke *R*² and AUC between SRH-only and combined-indicator models. Sensitivity analyses retained the full complete-case sample and reclassified ‘fair’ responses as poor/unhealthy or good/healthy.

**Results:**

In the primary high-contrast sample, 32.6% reported poor health in both domains, 20.2% poor oral health only, 7.6% poor general health only, and 39.6% good health in both domains. The poor – both groups had higher prevalence of mobility limitation, chronic conditions, depressive symptoms, and unmet healthcare needs. Among participants with good SRH, poor SROH identified hidden vulnerability, including greater mobility limitation, unmet medical needs, and chewing difficulty. For chewing difficulty, the combined-indicator model increased Nagelkerke *R*² from 0.373 to 0.540 and AUC from 0.822 to 0.883. Findings were directionally robust across sensitivity analyses.

**Conclusion:**

Combining SRH and SROH better identified hidden functional and care-related vulnerability than SRH alone, particularly among individuals reporting good general health. Its main value is subgroup identification for population health monitoring, not stand-alone clinical screening.

**Clinical relevance:**

Adding a single SROH item may help identify vulnerability missed by SRH alone, but clinical screening use requires further validation.

## Introduction

Self-rated health (SRH) is widely used to summarize how individuals perceive their overall health. Beyond objective medical conditions, it reflects day-to-day functioning and broader health experiences, including social relationships.^1^^,^^2^ Because SRH is a strong predictor of mortality and morbidity, it has been widely applied in epidemiological research and health policy evaluation as a simple, robust measure of population health.^3^, ^4^, ^5^

Nevertheless, SRH is a single-item, unstructured measure that compresses diverse experiences into one response, which can limit its ability to distinguish specific health domains. SRH also does not necessarily align with objective disease indicators; even among individuals with similar disease burdens, ratings may differ according to functional capacity, social participation, and cultural context.^5^^,^^6^ This heterogeneity indicates that SRH captures subjective interpretations of health that extend beyond clinical conditions alone.

Recent studies have reported that poor oral health is associated with lower overall SRH.^7^^,^^8^ Even so, many population-based studies continue to measure and analyse self-rated general health and self-rated oral health (SROH) as separate constructs. This separation may obscure discordance between perceived general and oral health, thereby overlooking groups experiencing multidimensional health vulnerability.

These observations suggest that general and oral health are not independent domains but are interconnected through shared functional, behavioural, and social pathways. This integration is consistent with the Common Risk Factor Approach, which emphasizes shared behavioural and social determinants across oral and general health, and with life-course perspectives that view oral health as shaped by cumulative exposures and functional trajectories over time.^9^^,^^10^ Such pathways may accumulate over time and shape how individuals perceive their overall health, indicating that integrating these domains may better capture multidimensional vulnerability.

This concern is especially salient in middle-aged and older adults. Even when systemic disease severity is modest, oral functional decline – such as impaired chewing – may constrain eating, interpersonal interactions, and social engagement.^11^^,^^12^ These functionally experienced limitations may not be captured by a single general health question. Together, this evidence suggests that subjective health perception may be more responsive to functional constraints than to the mere presence of diagnosed disease.

In parallel, global health policy increasingly frames oral health as an integral component of overall health, noncommunicable disease management, and universal health coverage.^13^^,^^14^ A recent Lancet analysis identified oral diseases as among the most prevalent conditions worldwide, affecting billions of people and closely linked to social and economic inequalities.^15^ The World Health Organization’s Global Strategy and Action Plan on Oral Health 2023 to 2030 similarly characterizes oral diseases as highly prevalent chronic conditions and calls for preventive, integrated approaches embedded within broader health systems.^16^ Collectively, these initiatives emphasize that oral health shapes nutrition, functional capacity, social participation, and quality of life, reinforcing its relevance to healthy ageing and comprehensive health promotion.^17^

This global framing aligns with policy directions in Korea, where community-based integrated care has been implemented to address population ageing and to coordinate medical, long-term care, and social services locally.^18^ However, monitoring and evaluation frameworks for these policies still rely largely on general health indicators, which may underrepresent complex vulnerability that includes oral health dimensions.^19^

Against this background, we used Korea Community Health Survey data to develop a combined indicator integrating self-rated general and oral health among middle-aged and older adults. We examined whether the combined indicator more comprehensively reflects individual- and community-level vulnerability. Specifically, we described the distribution of combined general–oral health categories and evaluated associated determinants. We also conducted sensitivity analyses using alternative classifications of the ‘fair’ response category to assess robustness.

## Methods

### Study population and data linkage

We analysed data from the 2024 Korea Community Health Survey (KCHS), which uses stratified, multistage cluster sampling and provides survey weights, strata, and primary sampling units. The original dataset included 231,728 participants. For this study, the population was restricted to adults aged ≥45 years; after excluding those younger than 45 years, 170,467 participants remained for the initial analysis.

For all analyses, we first defined a full complete-case sample among adults aged ≥45 years by excluding participants with missing data or refusal responses for SRH, SROH, covariates, or validation outcomes (*N* = 169,654). This full complete-case sample retained the entire SRH/SROH response spectrum, including all ‘fair’ responses.

**Fig. 1:**
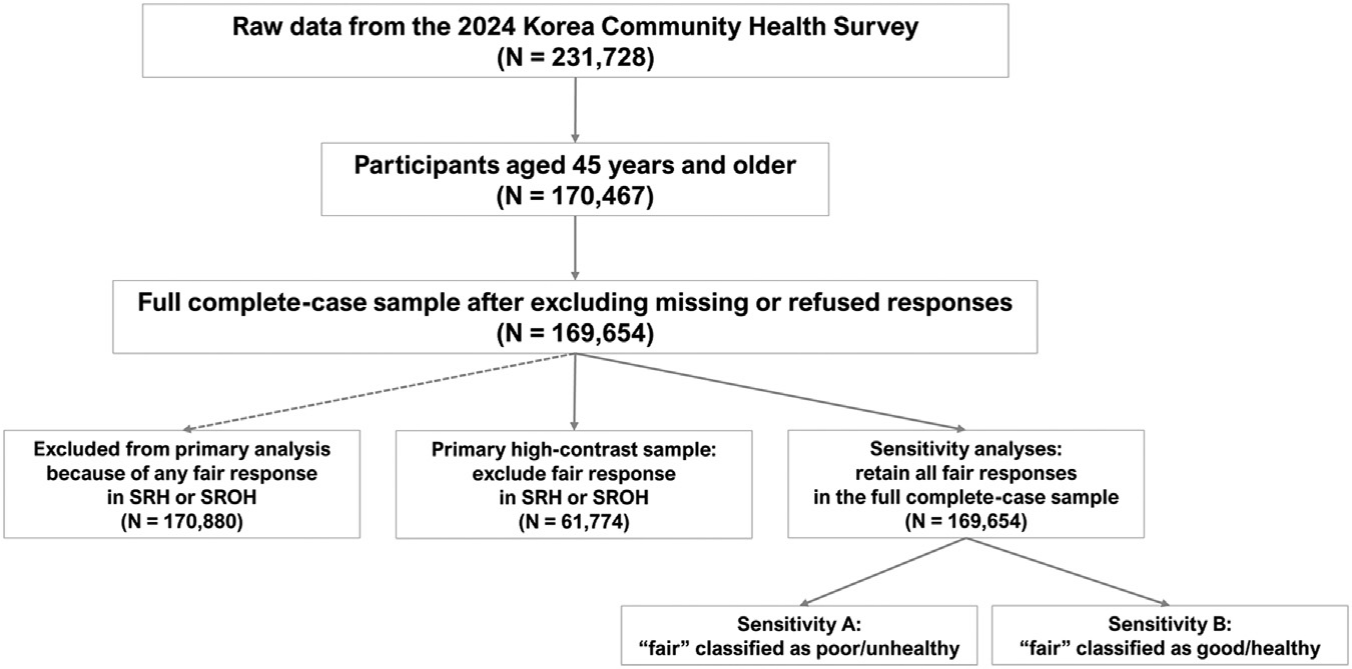
Flow diagram of participant selection from the 2024 Korea Community Health Survey (KCHS). Initial participants *N* = 231,728; adults aged 45 years and older *N* = 170,467; full complete-case sample after excluding missing or refused responses *N* = 169,654; participants excluded from the primary analysis because of any ‘fair’ response in SRH or SROH *N* = 107,880; primary high-contrast sample *N* = 61,774; sensitivity analyses retained all ‘fair’ responses in the full complete-case sample and classified them as either poor/unhealthy or good/healthy.

For the primary analysis, we used a high-contrast classification by excluding participants who selected ‘fair’ for either self-rated general health or SROH. This restriction was intended to maximize conceptual separation between clearly good and clearly poor perceived health states and resulted in a primary analytic sample of 61,774 individuals (Figure 1; Supplementary Table 1). Participants excluded from the primary analysis because of any ‘fair’ response in either SRH or SROH comprised 107,880 individuals. Because this approach may introduce selection towards more polarized responses, we compared the primary analytic sample with respondents excluded because of any ‘fair’ response, including effect-size measures (Supplementary Table 2).

To assess robustness, two sensitivity analyses were conducted using the full complete-case sample without excluding respondents with ‘fair’ responses (*N* = 169,654). In sensitivity analysis A, ‘fair’ responses in both SRH and SROH were classified as poor/unhealthy. In sensitivity analysis B, ‘fair’ responses were classified as good/healthy. Thus, the sensitivity analyses evaluated whether the main findings were robust when the full response spectrum was retained.

To incorporate community-level context, we linked external public administrative datasets to the KCHS data. Medically underserved area information was obtained from Korea Health Policy Research Institute reports, and urban classification variables were taken from supplementary KCHS materials. Depopulation area designation was based on Statistics Korea data, and integrated care pilot area status was identified from Ministry of Health and Welfare reports. Only 2024 external data were used. Variable definitions and classification criteria were verified before preprocessing, and community-level variables were matched one-to-one to individual records using municipal public health centre codes (Si/Gun/Gu [city/county/district] level) provided in the KCHS dataset.

### Variable definitions

#### Outcome variable

We constructed a combined health indicator by integrating self-rated general health and SROH into four categories: (1) good both (both general and oral health rated as good or very good); (2) poor oral health only (oral health rated as poor or very poor and general health rated as good or very good); (3) poor general health only (general health rated as poor or very poor and oral health rated as good or very good); and (4) poor both (both general and oral health rated as poor or very poor).

To enhance discriminant validity and conceptual clarity, the primary analysis used a strict good–poor contrast by excluding any ‘fair’ response in either SRH or SROH. Methodologically, the ‘fair’ category in SRH scales often functions as a heterogeneous ‘catch-all’ response, encompassing individuals with stable chronic conditions, those in transition, or those with varying sociocultural response styles.^20^^,^^21^ By focusing on the polarized ‘good’ and ‘poor’ categories, we aimed to minimize potential central tendency bias and reduce the risk of misclassification that can arise when intermediate health states are arbitrarily grouped.^22^ The included-vs-excluded comparison and full complete-case sensitivity analyses were used to assess robustness and contextualize representativeness (Supplementary Tables 2-7).

#### Independent variables and validation outcomes

Independent variables were classified as sociodemographic characteristics, health behaviours, health status and need factors, oral healthcare access variables, and community-level contextual factors. Chewing difficulty was not included as an independent variable in regression models to avoid conceptual circularity with SROH. Instead, chewing difficulty was treated as an external oral functional validation outcome.

Sociodemographic variables included sex, age, education level, monthly household income, marital status, and economic activity status. Household income was derived using both annual and monthly household income variables from the Korea Community Health Survey. Annual income was converted to a monthly equivalent where necessary, and the two variables were harmonized to construct a unified income measure. The resulting monthly household income was then categorized into five groups (<1, 1 to <2, 2 to <3, 3 to <4, and ≥4 million KRW) for analysis.

Health behaviours included smoking status and alcohol consumption. Health status and need factors included mobility limitation, hypertension (yes vs no), diabetes (yes vs no), depressive symptoms (yes vs no), perceived stress (yes vs no), and unmet healthcare needs (yes vs no).

Oral healthcare access variables included unmet dental care needs, dental scaling within the past year, toothbrushing after lunch, and toothbrushing after dinner or before bedtime. Community-level variables included residential area type (dong vs Eup/Myeon), urban classification (special metropolitan city, metropolitan city, city with <300,000 population, urban–rural mixed city, county, and county with a public medical institution), integrated care pilot area (yes vs no), medically underserved area (yes vs no), and depopulation area (not designated, at-risk or declining area, and area of concern).^23^ These variables were linked at the Si/Gun/Gu (municipal) level based on the administrative jurisdiction of public health centres as defined in the Korea Community Health Survey report.

#### Statistical analysis

We examined the distribution of the outcome across participant characteristics using complex-sample cross-tabulations. These cross-tabulations were used to describe unadjusted patterns, whereas multinomial regression models were used to estimate adjusted associations. We then performed survey-weighted multinomial logistic regression with the four-category combined indicator as the dependent variable, using ‘good both’ as the reference group. All regression analyses used the complex survey design information provided by the KCHS, including sampling weights, stratification variables, and primary sampling units.

Multivariable models were built hierarchically according to a conceptual framework informed by the social determinants of health, the Common Risk Factor Approach, life-course perspectives on oral health, and prior evidence on subjective health, oral healthcare access, and oral function.^5^^,^^9^^,^^10^^,^^24^, ^25^, ^26^, ^27^, ^28^, ^29^, ^30^, ^31^ Model 4 (base) included sociodemographic, behavioural, health status and need, and oral healthcare access variables as baseline adjustment variables. Model 5 (regional context) further incorporated community-level contextual variables to assess whether area-level conditions contributed beyond individual-level factors. Primary results are presented for Model 5, and the base model is provided in Supplementary Table 8.

Community-level variables were entered as fixed contextual covariates because the primary objective was to estimate population-averaged associations and to adjust for broad contextual conditions rather than to partition between-community variance. Accordingly, community-level associations should be interpreted descriptively and not as estimates of contextual variance components.

To avoid circularity, chewing difficulty was excluded from multinomial regression models and examined separately as an external validation outcome. The practical implications of discordant classifications were assessed by summarizing survey-weighted percentages of mobility limitation, unmet medical needs, and chewing difficulty among individuals with good SRH but poor SROH compared with those with good health in both domains (Table 4). Parallel hidden-vulnerability summaries were additionally calculated in the full complete-case sensitivity samples (Supplementary Table 7).

Incremental validity was assessed by comparing SRH-only models with combined-indicator models for each external validation outcome (mobility limitation, unmet medical needs, and chewing difficulty). For each outcome, the first model included SRH only, and the second model included the four-category combined SRH–SROH indicator. Nagelkerke *R*², AUC with 95% confidence intervals, Δ*R*², and ΔAUC were reported (Table 3).

Descriptive analyses and data preprocessing were performed using IBM SPSS Statistics version 25.0 (IBM Corp.). Survey-weighted multinomial logistic regression and AUC calculations were conducted in R version 4.5.1 (Posit) using relevant packages for complex survey data analysis.

Multicollinearity among independent variables was assessed using variance inflation factors (VIF). VIF values ranged from 1.090 to 4.011, with the maximum observed for urban–rural classification, indicating no problematic multicollinearity under the conventional threshold of 5. Detailed VIF values are provided in Supplementary Table 9.

We used a complete-case approach (listwise deletion) and did not impute missing values. Because exclusions due to ‘fair’ responses or missing/refusal responses could introduce selection bias, the primary high-contrast estimates should be interpreted as representing a deliberately polarized comparison rather than the full spectrum of perceived health.

#### Ethics statement

The KCHS is conducted by the Korea Disease Control and Prevention Agency (KDCA) under national ethical oversight, and anonymized raw data are publicly available for public research use.^23^ The study was approved by the Institutional Review Board of Yonsei University (IRB No. 1041849-202602-SB-032-01; 2026-03-05).

## Results

### Distribution of the combined self-rated general and oral health indicator

**Fig. 2:**
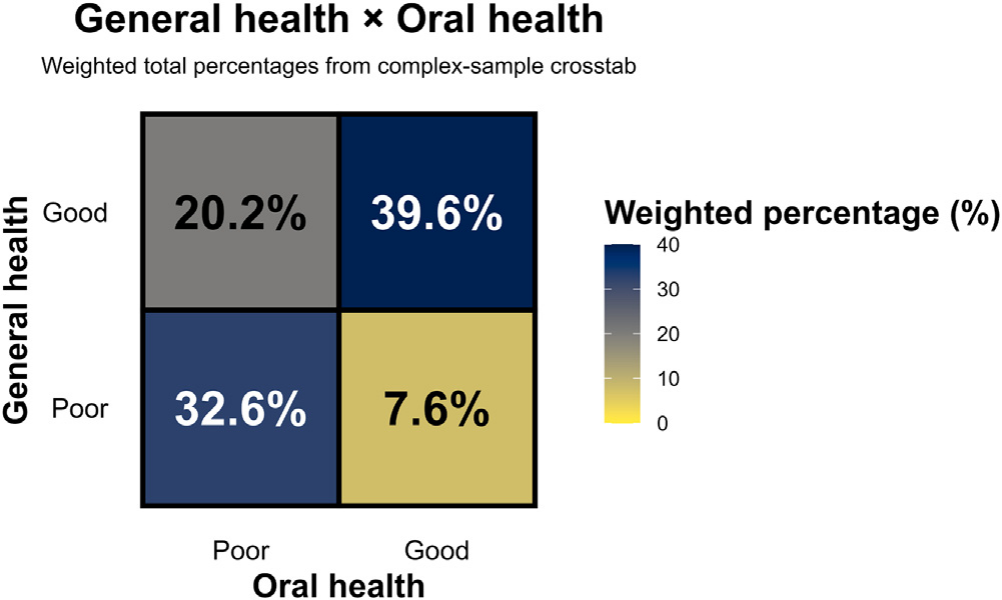
Survey-weighted distribution (%) of the four-group combined indicator integrating self-rated general health (SRH) and self-rated oral health (SROH) among adults aged 45 years and older in the 2024 KCHS primary high-contrast sample (*n* = 61,774). Groups: good both, poor oral only, poor general only, and poor both.

In the primary high-contrast sample (*n* = 61,774), the largest proportion of participants reported both good general and oral health (‘good both’; 39.6%), followed by poor health in both domains (‘poor both’; 32.6%), poor oral health only (20.2%), and poor general health only (7.6%) (Table 1; Figure 2). Relative to other groups, participants in the ‘poor both’ category had significantly higher proportions of mobility limitation, chronic conditions, depressive symptoms, and high perceived stress (Table 1; all *P* < .001).

**Table 1:** Survey-weighted distribution of the four combined self-rated general and oral health groups.

					Units: weighted %		
		*n* (unweighted)	Good both	Poor oral only	Poor general only	Poor both	*P* value
Total		61,774	39.6	20.2	7.6	32.6	
**Sociodemographic factors**							
Sex	Male	28,138	40.3	25.2	5.9	28.6	<.001
	Female	33,636	39.0	15.3	9.1	36.6	
Age (y)	45-54	10,809	59.6	21.3	5.6	13.6	<.001
	55-64	15,804	46.9	23.9	6.3	22.9	
	65-74	16,389	32.8	20.3	9.2	37.8	
	≥75 y	18,772	13.8	13.9	9.8	62.5	
Education level	≤Elementary school	20,841	10.9	13.2	10.2	65.7	<.001
	Middle school	8768	24.9	21.0	9.3	44.8	
	High school	17,507	42.9	23.4	7.5	26.1	
	≥College	14,658	60.7	21.2	5.2	12.8	
Household income (million KRW)	<1M	12,632	9.2	11.0	9.3	70.5	<.001
	1 to <2M	11,925	19.1	16.9	10.1	53.9	
	2 to <3M	8703	32.5	21.8	9.2	36.5	
	3 to <4M	6993	41.8	23.4	8.2	26.6	
	≥4M	21,521	56.7	22.6	5.6	15.2	
Spouse	With	41,835	45.8	21.8	7.1	25.4	<.001
	Without	19,939	24.2	16.2	8.8	50.8	
Economic activity	Yes	33,654	51.5	25.0	5.5	18.0	<.001
	No	28,120	24.9	14.1	10.1	50.8	
**Health behaviours**							
Smoking	Yes	9264	30.7	30.4	5.5	33.4	<.001
	No	52,510	41.4	18.1	8.0	32.5	
Drinking	Yes	30,527	49.0	24.0	5.6	21.4	<.001
	No	31,247	27.4	15.1	10.1	47.4	
**Health status and need factors**							
Mobility limitation	Yes	18,749	4.1	6.4	12.7	76.9	<.001
	No	43,025	50.2	24.3	6.1	19.5	
History of hypertension diagnosis	Yes	27,640	26.7	15.6	10.3	47.5	<.001
	No	34,134	48.0	23.1	5.8	23.1	
History of diabetes diagnosis	Yes	12,800	15.8	12.6	12.0	59.7	<.001
	No	48,974	45.0	21.9	6.6	26.6	
Depression	Yes	5651	12.8	9.4	11.5	66.3	<.001
	No	56,123	42.3	21.2	7.2	29.2	
Stress	Yes	12,601	22.7	14.1	10.6	52.6	<.001
	No	49,173	44.2	21.8	6.7	27.2	
Unmet medical needs	Yes	3218	16.2	15.6	7.4	60.8	<.001
	No	58,556	40.8	20.4	7.6	31.2	
**Oral healthcare access and oral function**							
Unmet dental needs	Yes	8950	10.2	29.1	3.2	57.5	<.001
	No	52,824	44.3	18.8	8.3	28.7	
Dental scaling in the past year	Yes	26,728	52.0	19.5	8.1	20.4	<.001
	No	35,046	26.5	20.9	7.0	45.6	
Toothbrushing after lunch	Yes	36,596	45.6	19.6	7.4	27.4	<.001
	No	25,178	29.4	21.2	7.8	41.6	
Toothbrushing after dinner/before bedtime	Yes	57,140	41.1	20.3	7.6	31.0	<.001
	No	4634	16.2	17.3	7.1	59.4	
Chewing difficulty	Present	21,064	1.8	24.6	1.3	72.2	<.001
	Absent	40,710	54.7	18.4	10.0	16.9	
**Community-level contextual factors**							
Residential area	Dong	29,922	42.1	20.1	7.5	30.3	<.001
	Eup/Myeon	31,852	31.3	20.4	7.9	40.4	
Urban-rural classification	Special metropolitan city (Gu)	5095	47.8	18.6	7.4	26.2	<.001
	Metropolitan city (Gu)	20,854	40.5	20.6	7.3	31.5	
	City with <300,000 population	2124	41.9	18.9	7.7	31.4	
	Urban-rural mixed city	10,304	33.2	20.7	8.2	37.9	
	County	18,785	31.8	20.2	7.9	40.1	
	County with a public medical institution	4612	27.5	19.3	8.8	44.4	
Integrated care pilot area	Yes	25,115	37.6	20.2	7.4	34.8	<.001
	No	36,659	40.6	20.1	7.6	31.7	
Medically underserved area	Yes	28,432	29.9	19.7	8.6	41.8	<.001
	No	33,342	41.4	20.2	7.4	31.0	
Depopulation area	Not designated	31,445	41.8	20.4	7.4	30.4	<.001
	At-risk or declining area	26,267	28.9	19.3	8.4	43.5	
	Area of concern	4062	34.5	18.4	7.7	39.5	

*n* values are unweighted counts; percentages are survey-weighted. *P* values account for the complex survey design.

M, million KRW.

Participants with unmet healthcare needs were more likely to report poor health in both domains. As shown in Figure 1, the full complete-case sample included 169,654 participants after excluding missing or refused responses. The primary high-contrast analysis then excluded respondents with any ‘fair’ response in either SRH or SROH, resulting in a final analytic sample of 61,774 participants. Respondents excluded from the primary analysis because of any ‘fair’ response comprised 107,880 individuals. Similar distribution patterns were observed in Sensitivity Analyses A and B, which retained all fair responses in the full complete-case sample (*N* = 169,654) and classified them as either poor/unhealthy or good/healthy, respectively.

### Chewing difficulty as an external functional validation outcome

Chewing difficulty, an indicator of oral functional status, differed markedly across combined health categories. Participants reporting chewing difficulty were most frequently classified in the ‘poor both’ group, with proportions exceeding those in the ‘poor general health only’ and ‘poor oral health only’ groups (Table 1).

In survey-weighted multinomial logistic regression, factors associated with combined health status were examined after excluding chewing difficulty to avoid circularity (Table 2). In the fully adjusted model, mobility limitation showed the strongest association with the ‘poor both’ group compared with the ‘good both’ group (AOR 12.8, 95% CI 11.4-14.4), while unmet dental needs were strongly associated with both the ‘poor oral only’ and ‘poor both’ groups (AOR 5.9, 95% CI 5.3-6.7; and AOR 6.1, 95% CI 5.4-7.0, respectively). Chewing difficulty was subsequently treated as an external validation outcome, and its distribution across combined health categories remained consistent with the primary findings, with the highest prevalence observed in the ‘poor both’ group (Table 4).

**Table 2:** Survey-weighted multinomial logistic regression of the four combined self-rated general and oral health groups (Model 5, chewing difficulty excluded).

Variable	Category (reference)	Poor oral only vs good both, AOR (95% CI)	Poor general only vs good both, AOR (95% CI)		Poor both vs good both, AOR (95% CI)	*P* value
**Sociodemographic factors**						
Sex	Female	0.7 (0.6-0.7)	1.1 (1.0-1.2)		0.8 (0.7-0.9)	<.001
Age (y)	55-64	1.3 (1.2-1.4)	0.9 (0.8-1.0)		1.2 (1.1-1.4)	
	65-74	1.3 (1.2-1.5)	0.8 (0.7-1.0)		1.1 (1.0-1.3)	<.001
	≥75	1.7 (1.5-1.9)	0.8 (0.6-0.9)		1.3 (1.1-1.5)	
Education level	≤Elementary school	2.3 (2.0-2.6)	2.7 (2.3-3.2)		4.6 (4.0-5.2)	<.001
	Middle school	1.8 (1.6-2.0)	2.0 (1.7-2.3)		2.9 (2.6-3.3)	
	High school	1.3 (1.2-1.4)	1.5 (1.3-1.7)		1.8 (1.6-1.9)	
Household income (million KRW)	<1M	1.5 (1.3-1.7)	2.0 (1.7-2.4)		2.9 (2.6-3.4)	<.001
	1 to <2M	1.3 (1.1-1.4)	1.7 (1.5-2.0)		2.2 (1.9-2.4)	
	2 to <3M	1.2 (1.1-1.4)	1.5 (1.3-1.8)		1.7 (1.5-1.9)	
	3 to <4M	1.1 (1.0-1.3)	1.3 (1.2-1.6)		1.4 (1.2-1.5)	
Spouse	Without	1.0 (0.9-1.1)	1.1 (1.0-1.2)		1.2 (1.1-1.3)	<.01
Economic activity	No	1.0 (1.0-1.1)	2.0 (1.8-2.2)		2.2 (2.1-2.4)	<.001
Health behaviours						
Smoking	Yes	1.8 (1.7-2.0)	1.3 (1.1-1.5)		1.8 (1.6-2.0)	<.001
Drinking	Yes	1.0 (0.9-1.0)	0.6 (0.5-0.6)		0.6 (0.5-0.6)	<.001
**Health status and need factors**						
Mobility limitation	Yes	2.0 (1.8-2.3)	10.6 (9.2-12.2)		12.8 (11.4-14.4)	<.001
History of hypertension diagnosis	Yes	0.9 (0.8-1.0)	1.8 (1.6-1.9)		1.6 (1.5-1.7)	<.001
History of diabetes diagnosis	Yes	1.3 (1.2-1.5)	3.6 (3.2-4.0)		4.1 (3.7-4.5)	<.001
Depression	Yes	1.2 (1.1-1.5)	2.9 (2.4-3.4)		3.3 (2.8-3.8)	<.001
Stress	Yes	1.3 (1.1-1.4)	3.4 (3.0-3.8)		4.0 (3.6-4.4)	<.001
Unmet medical needs	Yes	1.1 (0.9-1.4)	2.0 (1.5-2.5)		2.2 (1.8-2.7)	<.001
**Oral healthcare access and oral function**						
Unmet dental needs	Yes	5.9 (5.3-6.7)	1.4 (1.1-1.7)		6.1 (5.4-7.0)	<.001
Dental scaling in the past year	No	1.3 (1.3-1.4)	0.9 (0.8-1.0)		1.5 (1.4-1.6)	<.001
Toothbrushing after lunch	No	1.2 (1.1-1.3)	1.2 (1.1-1.3)		1.3 (1.2-1.4)	<.001
Toothbrushing after dinner/before bedtime	No	1.2 (1.0-1.4)	1.3 (1.0-1.6)		1.6 (1.4-1.9)	<.001
**Community-level contextual factors**						
Residential area	Eup/Myeon	0.9 (0.9-1.1)	1.0 (0.9-1.2)		0.9 (0.8-1.1)	0.388
Urban-rural classification	Metropolitan city (Gu)	1.3 (1.1-1.4)	1.3 (1.2-1.5)		1.6 (1.4-1.8)	<.001
	City with <300,000 population	1.1 (0.9-1.3)	1.3 (1.0-1.7)		1.4 (1.1-1.7)	
	Urban-rural mixed city	1.4 (1.2-1.5)	1.5 (1.2-1.8)		1.6 (1.4-1.9)	
	County	1.1 (0.9-1.3)	1.2 (0.9-1.5)		1.3 (1.1-1.5)	
	County with a public medical institution	1.1 (0.9-1.4)	1.3 (1.0-1.8)		1.4 (1.1-1.7)	
Integrated care pilot area	No	0.9 (0.9-1.0)	1.0 (0.9-1.1)		0.9 (0.8-1.0)	<0.05
Medically underserved area	Yes	1.0 (0.9-1.1)	1.1 (0.9-1.3)		0.9 (0.8-1.0)	<0.05
Depopulation area	At-risk or declining area	1.0 (0.9-1.1)	1.0 (0.9-1.2)		1.1 (1.0-1.3)	<0.05
	Area of concern	0.9 (0.8-1.0)	0.9 (0.8-1.1)		1.1 (0.9-1.2)	

Reference categories: male; age 45 to 54 years; ≥college education; household income ≥4 million KRW; living with spouse; economically active; smoking (No); drinking (No); mobility limitation (No); hypertension (No); diabetes diagnosis (No); depression (No); stress (No); no unmet medical needs; no unmet dental needs; dental scaling within the past year (Yes); toothbrushing after lunch (Yes); toothbrushing after dinner or before bedtime (Yes); residential area – dong; urban–rural classification – special metropolitan city (Gu); integrated care pilot area (Yes); medically underserved area (No); depopulation area (Not designated). Model 4 (base): sociodemographic, behavioural, health status and need, and oral healthcare access variables. Model 5 (regional context): Model 4 + community-level contextual variables. Chewing difficulty is excluded from all regression models and used only as an external validation outcome.

Units: AOR, adjusted odds ratio; 95% CI, confidence interval.

### Social gradient and oral healthcare access factors

Across multivariable models, older age, lower educational attainment, lower household income, and economic inactivity were independently associated with poorer combined health status (Table 2; Supplementary Table 8), indicating a clear social gradient in perceived general–oral health vulnerability. Smoking was associated with poorer combined health status, whereas current drinking showed inverse associations with poor general health only and poor both groups; however, these findings should be interpreted cautiously given the potential for healthy-drinker and sick-quitter bias in cross-sectional studies.

Oral healthcare access factors were also associated with combined health status. Participants reporting unmet dental care needs, no dental scaling within the past year, and lower adherence to toothbrushing after lunch or after dinner/before bedtime were more likely to belong to poorer combined health categories.

### Consistency across sensitivity analyses

Sensitivity Analyses A and B, which retained the full complete-case sample and reclassified the ‘fair’ response category in opposite directions, produced findings generally consistent with the primary analysis. In both analyses, key correlates – including mobility limitation, unmet dental needs, lower educational attainment, lower household income, and economic inactivity – remained associated with poorer combined health status in the same direction (Supplementary Tables 3-6). These concordant findings support the robustness of the primary results despite alternative classifications of the ‘fair’ category (Table 5).

### Incremental validity and practical relevance

Compared with models using SRH alone, models incorporating the combined SRH–SROH indicator showed limited improvement in discrimination for mobility limitation and unmet medical needs (Table 3). For mobility limitation, the combined indicator improved model fit (Nagelkerke *R*², 0.577 vs 0.507; Δ*R*² = +0.070), but discrimination changed only minimally (ΔAUC = +0.001). Similarly, no meaningful improvement was observed for unmet medical needs (Δ*R*² = 0.000; ΔAUC = 0.000).

**Table 3:** Incremental validity and discrimination for external validation outcomes (*N* = 61,774).

External validation outcome	Model	Nagelkerke *R*²	AUC (95% CI)	Δ*R*²	ΔAUC
**Mobility limitation**	SRH-only model	0.507	0.905 (0.903-0.908)	Ref	Ref
	Combined SRH–SROH indicator	0.577	0.906 (0.904-0.909)	+0.070	+0.001
**Unmet medical needs**	SRH-only model	0.188	0.793 (0.784-0.801)	Ref	Ref
	Combined SRH–SROH indicator	0.188	0.793 (0.785-0.801)	0.000	0.000
**Chewing difficulty**	SRH-only model	0.373	0.822 (0.818-0.825)	Ref	Ref
	Combined SRH–SROH indicator	0.540	0.883 (0.880-0.885)	+0.167	+0.061

Each binary validation outcome was compared using an SRH-only model and a combined SRH–SROH indicator model. Nagelkerke *R*² and AUC were estimated with survey weights. Chewing difficulty was evaluated as an external oral functional validation outcome and was not included as a covariate in the multinomial regression models.

AUC, area under the curve; CI, confidence interval; SRH, self-rated health; SROH, self-rated oral health.

In contrast, the combined indicator substantially improved discrimination and explained variance for chewing difficulty, an external oral functional validation outcome. Compared with the SRH-only model, the combined-indicator model increased Nagelkerke *R*² from 0.373 to 0.540 and improved AUC from 0.822 (95% CI 0.818-0.825) to 0.883 (95% CI 0.880-0.885), corresponding to Δ*R*² = +0.167 and ΔAUC = +0.061.

These findings suggest that the added value of the combined indicator lies less in improving prediction of general vulnerability outcomes than in identifying oral functional vulnerability that may not be captured by SRH alone.

### Vulnerability among individuals reporting good general health

Among individuals reporting good general health in the primary high-contrast sample, those reporting poor oral health had significantly higher prevalence of mobility limitation (7.4% vs 2.4%), unmet medical needs (3.7% vs 2.0%), and chewing difficulty (34.8% vs 1.3%) than those reporting good health in both domains (Table 4; all *P* < .001). These findings suggest that poor SROH may identify substantial functional and healthcare-related vulnerability even among individuals who otherwise perceive their general health as good.

**Table 4:** Vulnerability among individuals reporting good self-rated general health.

Outcome	Good both (weighted %)	Poor oral only (weighted %)	*P* value
Mobility limitation	2.4	7.4	<.001
Unmet medical needs	2.0	3.7	<.001
Chewing difficulty	1.3	34.8	<.001

Values are survey-weighted percentages. This table uses the primary high-contrast sample.

SRH, self-rated health; SROH, self-rated oral health.

**Table 5:** Robustness summary across primary and full complete-case sensitivity analyses.

Key correlate	Contrast	Primary high-contrast sample, AOR (95% CI)	Sensitivity A: fair = poor/unhealthy, AOR (95% CI)	Sensitivity B: fair = good/healthy, AOR (95% CI)
**Mobility limitation**	Poor both vs good both	12.8 (11.4-14.4)	5.2 (4.7-5.8)	6.1 (5.8-6.5)
**Unmet dental needs**	Poor both vs good both	6.1 (5.4-7.0)	3.8 (3.4-4.2)	2.9 (2.7-3.2)
**Unmet medical needs**	Poor both vs good both	2.2 (1.8-2.7)	1.7 (1.5-2.0)	1.7 (1.5-1.9)
**Education ≤elementary school**	Poor both vs good both	4.6 (4.0-5.2)	2.7 (2.4-2.9)	3.1 (2.8-3.4)
**Household income <1M KRW**	Poor both vs good both	2.9 (2.5-3.4)	1.9 (1.7-2.1)	2.2 (2.1-2.4)
**Economic inactivity**	Poor both vs good both	2.2 (2.1-2.4)	1.4 (1.3-1.5)	2.0 (1.9-2.2)
**Current smoking**	Poor both vs good both	1.8 (1.6-2.0)	1.5 (1.4-1.6)	1.5 (1.4-1.6)
**Current drinking**	Poor both vs good both	0.6 (0.5-0.6)	0.9 (0.8-0.9)	0.6 (0.5-0.6)

Sensitivity analyses A and B used the full complete-case sample among adults aged ≥45 years without excluding respondents with ‘fair’ responses. Full regression results are provided in Supplementary Tables 4 and 6. Current drinking should be interpreted descriptively because inverse associations may reflect healthy-drinker or sick-quitter bias.

## Discussion

Our findings indicate that integrating self-rated general health and SROH into a combined indicator provides a broader representation of multidimensional vulnerability at both individual and community levels. Participants who perceived both domains as poor were disproportionately affected by mobility limitation, chronic diseases, depressive symptoms, and unmet healthcare needs. These associations remained robust after adjustment for individual and contextual determinants, while chewing difficulty was examined separately as an external validation outcome. Importantly, the same overall pattern was observed in sensitivity analyses that retained the full complete-case sample (*N* = 169,654) and reclassified ‘fair’ responses in opposite directions, which argues against the primary good–poor contrast merely creating spurious differences. The gain in model fit over SRH alone was modest for general vulnerability outcomes, and discrimination changed little, indicating that the main added value of SROH is not a large statistical increase in prediction but the identification of clinically and policy-relevant discordance. This interpretation is supported by recent evidence showing that delayed dental care reflects broader disparities in healthcare access and utilization, highlighting the importance of identifying vulnerable subgroups beyond conventional indicators.^24^ In that sense, the combined indicator may be most useful for identifying hidden multidimensional vulnerability, rather than for improving global discrimination performance.

A central practical advantage of the combined indicator was its ability to uncover hidden vulnerability among individuals who would be classified as healthy by self-rated general health alone. Among those reporting good general health, participants reporting poor oral health had markedly higher prevalence of mobility limitation (7.4% vs 2.4%), unmet medical needs (3.7% vs 2.0%), and especially chewing difficulty (34.8% vs 1.3%) than those reporting good health in both domains. These patterns suggest that SRH alone may underrecognize vulnerability related to functional impairment and oral health-related limitations. Rather than substantially changing discrimination, the combined indicator adds practical value by revealing vulnerability that may remain concealed in single-domain population monitoring.

Prior work has shown that poorer overall health is associated with more frequent reports of oral health problems.^25^ Among community-dwelling older adults, depressive symptoms and eating limitations attributable to oral conditions have been linked to poorer self-rated general health, even when general and oral health are analysed separately.^26^ Population-based studies also indicate that oral health problems are independently associated with poorer subjective health perception, particularly through pathways involving functional limitation and mental health factors.^27^ Our results align with this literature and suggest that oral health contributes meaningfully to overall health perception beyond systemic disease burden alone.

Functional limitations, including chewing difficulty, emerged as important features of poor combined health status. These patterns showed clear differences across combined health categories and are consistent with prior evidence linking oral functional impairment to nutritional compromise, impaired activities of daily living, reduced social participation, and diminished quality of life.^28^, ^29^, ^30^, ^31^

Accordingly, such functional limitations may serve as early indicators of frailty and functional decline in ageing populations. In this context, oral functional impairment may help explain how oral health relates to broader health perception, particularly among individuals who otherwise report good general health.

Because these limitations can coexist with otherwise favourable general health ratings, paired assessment of SRH and SROH may be preferable to SRH alone when identifying early functional vulnerability in middle-aged and older adults. Consistent with this, recent multilevel evidence from Korea indicates that mobility limitations are strongly associated with unmet dental needs, underscoring the role of functional constraints in shaping oral health–related vulnerability.^32^ Contextual associations were selective rather than uniform. After adjustment for individual-level determinants, with chewing difficulty evaluated separately as an external validation outcome, urban–rural classification remained associated with combined health status, whereas residential area, integrated care pilot area, and medically underserved area generally did not show clear independent associations in the fully adjusted model. This pattern suggests that, within the available measures, individual functional status exerted a more dominant influence than broad regional context in distinguishing combined health vulnerability. At the same time, broad administrative indicators may capture structural context only imperfectly and may be less sensitive to the everyday access barriers, service quality, or social environments that shape subjective health perception.

Because our analyses were conducted using survey-weighted models rather than multilevel approaches, we did not explicitly partition variance across individual and community levels.

Nevertheless, the persistence of some regional differences is consistent with multilevel evidence that community fiscal capacity, social welfare expenditure, and dental infrastructure are independently associated with SROH among Korean adults.^33^ Together, these findings suggest that community-level context matters, but its influence may be uneven and may require more granular measures than those available in the present study.

Future studies using multilevel models may provide additional insight into between-community variation and contextual effects.

Integrating general and oral health perceptions also highlighted discordance that may not be apparent using a single-item general health measure. While subjective health is often used to predict long-term outcomes such as mortality and morbidity, our findings suggest that it may also serve as a cross-sectional marker of multidimensional vulnerability when interpreted using a combined-domain framework. The discordance observed – especially among individuals reporting good general but poor oral health – appears to reflect meaningful functional and healthcare-related disadvantages rather than measurement inconsistency. Incongruence between general and oral health perceptions may therefore capture lived functional experiences and social context rather than measurement error,^34^^,^^35^ offering insight into complex health states that conventional indicators may miss.

These results have implications for health monitoring and policy. Global frameworks increasingly emphasize oral health as integral to overall health and noncommunicable disease strategies,^36^ and the global burden of oral disease remains strongly patterned by social inequality.^15^ Combining general and oral health perceptions may therefore improve identification of structurally and functionally vulnerable populations, particularly in ageing societies. At the current stage, the combined SRH–SROH indicator may be most appropriate as a population-monitoring and subgroup-identification tool rather than as a stand-alone clinical screening instrument. Its clinical use should be evaluated in future studies using prospective outcomes and formal reclassification metrics.

Several limitations should be considered. First, the cross-sectional design precludes causal inference regarding directionality between general and oral health perceptions. Second, both SRH and SROH were measured with single self-reported items; although practical for surveillance, they may not fully capture objective oral conditions such as tooth loss, number of remaining teeth, prosthetic status, or periodontal disease. These objective oral health measures were not available for inclusion in the present analytic model, and responses may also reflect sociocultural differences in interpretation. Third, the primary analysis reduced the full complete-case sample from 169,654 to 61,774 by excluding respondents with ‘fair’ response in either SRH or SROH after listwise deletion. Supplementary Table 2 indicates that respondents excluded at this final step tended to be younger and more socioeconomically advantaged than the final analytic sample, so selection related to the strict good–poor contrast remains possible. However, sensitivity analyses that reclassified ‘fair’ responses and retained the full complete-case sample (*N* = 169,654) yielded directionally consistent findings, which mitigates but does not eliminate this concern. Fourth, excluding the ‘fair’ category improved conceptual contrast but may limit generalizability to the broader population in which intermediate responses are common. At the same time, the ‘fair’ category in SRH is known to represent a heterogeneous group, potentially including individuals in transitional states or at risk of early frailty. In this context, the exclusion of ‘fair’ responses should be understood as an intentional analytic strategy aimed at maximizing discriminant validity by focusing on a clear contrast between good and poor health states. Finally, community-level variables were linked at the municipal level and may not capture neighbourhood-scale environmental variation, and sex-stratified analyses were not performed.

Overall, these findings support the conceptual and empirical value of integrating oral and general health perceptions into a multidimensional indicator of vulnerability, while also indicating the need for further validation across the full response spectrum.

## Conclusion

Compared with SRH alone, a combined indicator integrating self-rated general and oral health may provide additional insight into hidden functional and care-related vulnerability, particularly among individuals who report good general health but poor oral health.

The added value of the combined indicator appears to lie less in large gains in overall discrimination than in identifying discordant risk profiles that may otherwise be overlooked. These patterns were robust to alternative reclassification of common intermediate (‘fair’) responses. Incorporating oral health perception into population health monitoring may therefore support more integrated approaches to identifying vulnerable subgroups, although further evaluation using discrimination-based and reclassification metrics is needed before considering its use in clinical screening.

## Author contributions

H.-N.K. contributed to the conception and design of the study, data curation, formal analysis, interpretation of the findings, and drafting of the manuscript. S.K. contributed to the conception of the study, interpretation of the findings, and critical revision of the manuscript for important intellectual content. N.-H.K. contributed to the conception and design of the study, interpretation of the findings, supervision of the study, and critical revision of the manuscript for important intellectual content. All authors materially participated in the work, approved the final version of the manuscript, and agree to be accountable for all aspects of the work.

## Declaration of generative AI and AI-assisted technologies in the writing process

During the preparation of this work, the authors used ChatGPT (GPT-5OpenAI) for language refinement and to improve clarity of expression. All AI-assisted content was reviewed, edited, and verified by the authors, who take full responsibility for the final manuscript.

## Conflict of interest

The authors declare that they have no known competing financial interests or personal relationships that could have appeared to influence the work reported in this article.

## Appendix Supplementary materials

Image, application 1

## Footnotes

Supplementary material associated with this article can be found in the online version at doi:10.1016/j.identj.2026.109729.
