# Supplementary material for: Validity of a Combined General and Oral Health Indicator for Vulnerability
Source: Int Dent J. 2026 Jul 2;76(5):109729. doi: 10.1016/j.identj.2026.109729 (PMC13355170; doi:10.1016/j.identj.2026.109729)
Supplement: Supplementary file 1 [file mmc1.docx]

**List of Supplementary Tables**

**Supplementary Table 1. Participant-flow counts and analytic sample definitions**

**Supplementary Table 2. Included-versus-excluded comparison with effect sizes**

**Supplementary Table 3. Sensitivity Analysis A distribution: fair classified as poor/unhealthy**

**Supplementary Table 4. Sensitivity Analysis A multinomial regression: fair classified as poor/unhealthy**

**Supplementary Table 5. Sensitivity Analysis B distribution: fair classified as good/healthy**

**Supplementary Table 6. Sensitivity Analysis B multinomial regression: fair classified as good/healthy**

**Supplementary Table 7. Hidden-vulnerability outcomes across primary and sensitivity samples**

**Supplementary Table 8. Base model (Model 4) multinomial regression, chewing difficulty excluded**

**Supplementary Table 9. Variance inflation factor (VIF) diagnostics**

**Supplementary Table 1. Participant-flow counts and analytic sample definitions**

| **Step** | **n (unweighted)** | **Definition** |
| --- | --- | --- |
| **Raw KCHS 2024 participants** | 231,728 | Original dataset |
| **Adults aged ≥45 years** | 170,467 | Age-restricted source population |
| **Full complete-case sample after excluding missing/refused responses for SRH, SROH, covariates, or validation outcomes** | 169,654 | Full SRH/SROH response spectrum retained, including fair responses |
| **Excluded from primary analysis because of any fair response in SRH or SROH** | 107,880 | Respondents with fair responses in at least one of SRH or SROH |
| **Primary high-contrast sample** | 61,774 | Good/very good versus poor/very poor contrast |
| **Sensitivity Analysis A sample** | 169,654 | Fair classified as poor/unhealthy |
| Sensitivity Analysis B sample | 169,654 | Fair classified as good/healthy |

Note: SRH = self-rated health; SROH = self-rated oral health.

**Supplementary Table 2. Comparison of the primary high-contrast sample with complete-case respondents excluded because of any “fair” response**

| **Characteristic** | **Category** | **Primary sample n / weighted %** | **Excluded any-fair group n / weighted %** | **Absolute difference (percentage points)** | **Standardized difference** | **Overall effect size (Cramer’s V for variable)** |
| --- | --- | --- | --- | --- | --- | --- |
| Sex | Male | 28,138 (49.3%) | 47,879 (47.2%) | 2.1 | 0.042 | 0.011 |
|  | Female | 33,636 (50.7%) | 60,001 (52.8%) | 2.1 | 0.042 |  |
| Age (years) | 45-54 | 10,809 (26.2%) | 26,153 (33.3%) | 7.1 | 0.156 | 0.155 |
|  | 55-64 | 15,804 (28.6%) | 33,551 (32.2%) | 3.6 | 0.078 |  |
|  | 65-74 | 16,389 (23.1%) | 29,086 (22.0%) | 1.1 | 0.026 |  |
|  | ≥75 | 18,772 (22.1%) | 19,090 (12.6%) | 9.5 | 0.253 |  |
| Education level | ≤Elementary school | 20,841 (21.8%) | 23,981 (13.6%) | 8.2 | 0.216 | 0.128 |
|  | Middle school | 8,768 (12.7%) | 16,097 (11.9%) | 0.8 | 0.024 |  |
|  | High school | 17,507 (31.9%) | 37,667 (37.1%) | 5.2 | 0.110 |  |
|  | ≥College | 14,658 (33.7%) | 30,135 (37.4%) | 3.7 | 0.077 |  |
| Household income (million KRW) | <1M | 12,632 (13.2%) | 12,701 (7.2%) | 6.0 | 0.199 | 0.132 |
|  | 1-<2M | 11,925 (15.5%) | 17,712 (12.2%) | 3.3 | 0.096 |  |
|  | 2-<3M | 8,703 (13.1%) | 17,022 (13.6%) | 0.5 | 0.015 |  |
|  | 3-<4M | 6,993 (12.0%) | 14,380 (13.4%) | 1.4 | 0.043 |  |
|  | >=4M | 21,521 (46.2%) | 46,065 (53.6%) | 7.4 | 0.149 |  |
| Spouse | With | 41,835 (71.4%) | 80,878 (77.1%) | 5.7 | 0.134 | 0.078 |
|  | Without | 19,939 (28.5%) | 27,002 (22.9%) | 5.6 | 0.128 |  |
| Economic activity | Yes | 33,654 (55.4%) | 69,299 (63.6%) | 8.2 | 0.165 | 0.096 |
|  | No | 28,120 (44.6%) | 38,581 (36.4%) | 8.2 | 0.165 |  |
| Mobility limitation | Yes | 18,749 (22.9%) | 16,132 (11.0%) | 11.9 | 0.339 | 0.183 |
|  | No | 43,025 (77.1%) | 91,748 (89.0%) | 11.9 | 0.339 |  |
| Unmet medical needs | Yes | 3,218 (4.8%) | 4,388 (4.1%) | 0.7 | 0.037 | 0.027 |
|  | No | 58,556 (95.2%) | 103,492 (95.9%) | 0.7 | 0.037 |  |
| Unmet dental needs | Yes | 8,950 (13.6%) | 12,519 (11.6%) | 2.0 | 0.060 | 0.042 |
|  | No | 52,824 (86.4%) | 95,361 (88.4%) | 2.0 | 0.060 |  |
| Chewing difficulty | Present | 21,064 (28.5%) | 16,640 (13.2%) | 15.3 | 0.402 | 0.216 |
|  | Absent | 40,710 (71.5%) | 91,240 (86.8%) | 15.3 | 0.402 |  |

Notes: n values are unweighted counts; percentages are survey-weighted. The excluded group comprises complete-case respondents with any fair SRH or SROH response. Standardized differences are presented by category; Cramer’s V is presented for each variable.

| **Supplementary Table 3. Sensitivity Analysis A: survey-weighted distribution when fair responses were classified as poor/unhealthy** | | | | | | | |
| --- | --- | --- | --- | --- | --- | --- | --- |
| Characteristic | Category | **n (unweighted)** | **Good both (weighted %)** | **Poor oral only (weighted %)** | **Poor general only (weighted %)** | **Poor both (weighted %)** | **p-value** |
| Total | | 169,654 | 13.5 | 21.8 | 10.8 | 53.9 |  |
| **Sociodemographic factors** | | |  |  |  |  |  |
| Sex | Male | 76,017 | 14.1 | 25.1 | 9.7 | 51.1 | <0.001 |
|  | Female | 93,637 | 12.9 | 18.8 | 11.7 | 56.6 |  |
| Age (years) | 45–54 | 36,962 | 17.2 | 26.1 | 10.5 | 46.3 | <0.001 |
|  | 55–64 | 49,355 | 14.8 | 23.7 | 10.7 | 50.8 |  |
|  | 65–74 | 45,475 | 11.5 | 19.1 | 11.5 | 57.9 |  |
|  | ≥75 years | 37,862 | 6.5 | 13.7 | 10.4 | 69.4 |  |
| Education level | ≤Elementary school | 44,822 | 4.9 | 12.0 | 10.2 | 72.9 | <0.001 |
|  | Middle school | 24,865 | 8.8 | 17.6 | 11.2 | 62.4 |  |
|  | High school | 55,174 | 13.2 | 22.8 | 10.7 | 53.3 |  |
|  | ≥College | 44,793 | 19.3 | 26.7 | 11.0 | 43.1 |  |
| Household income  (million KRW) | <1M | 25,333 | 4.5 | 10.7 | 9.2 | 75.5 | <0.001 |
|  | 1–<2M | 29,637 | 7.6 | 15.4 | 10.9 | 66.2 |  |
|  | 2–<3M | 25,725 | 10.8 | 20.2 | 11.3 | 57.7 |  |
|  | 3–<4M | 21,373 | 13.2 | 23.4 | 11.0 | 52.4 |  |
|  | ≥4M | 67,586 | 17.5 | 25.5 | 10.8 | 46.2 |  |
| Spouse | With | 122,713 | 14.8 | 23.4 | 10.9 | 50.9 | <0.001 |
|  | Without | 46,941 | 9.5 | 17.1 | 10.3 | 63.1 |  |
| Economic activity | Yes | 102,953 | 16.0 | 25.4 | 10.3 | 48.2 | <0.001 |
|  | No | 66,701 | 9.7 | 16.2 | 11.4 | 62.7 |  |
| **Health behaviors** | | |  |  |  |  |  |
| Smoking | Yes | 25,771 | 10.4 | 25.2 | 8.6 | 55.9 | <0.001 |
|  | No | 143,883 | 14.1 | 21.1 | 11.2 | 53.5 |  |
| Drinking | Yes | 64,635 | 15.2 | 24.9 | 10.3 | 49.6 | <0.001 |
|  | No | 75,019 | 10.7 | 16.7 | 11.5 | 61.1 |  |
| **Health status and need factors** | | |  |  |  |  |  |
| Mobility limitation | Yes | 34,881 | 2.1 | 6.2 | 11.2 | 80.4 | <0.001 |
|  | No | 134,773 | 15.5 | 24.6 | 10.7 | 49.2 |  |
| History of hypertension diagnosis | Yes | 71,119 | 9.6 | 15.7 | 12.2 | 62.6 | <0.001 |
|  | No | 98,535 | 15.8 | 25.4 | 9.9 | 48.9 |  |
| History of diabetes diagnosis | Yes | 31,333 | 6.0 | 11.4 | 12.5 | 70.0 | <0.001 |
|  | No | 138,321 | 15.0 | 23.8 | 10.4 | 50.8 |  |
| Depression | Yes | 12,195 | 5.5 | 10.2 | 11.5 | 72.8 | <0.001 |
|  | No | 157,459 | 14.1 | 22.7 | 10.7 | 52.4 |  |
| Stress | Yes | 30,562 | 8.4 | 14.6 | 11.7 | 65.3 | <0.001 |
|  | No | 139,092 | 14.7 | 23.6 | 10.6 | 51.1 |  |
| Unmet medical needs | Yes | 7,606 | — | — | — | — | <0.001 |
|  | No | 162,048 | 13.8 | 22.1 | 10.9 | 53.2 |  |
| **Oral healthcare access and oral function** | | | |  |  |  |  |
| Unmet dental needs | Yes | 21,469 | 3.9 | 22.0 | 3.9 | 70.3 | <0.001 |
|  | No | 148,185 | 14.8 | 21.8 | 11.7 | 51.6 |  |
| Dental scaling in the past year | Yes | 82,248 | 16.5 | 23.1 | 12.2 | 48.2 | <0.001 |
|  | No | 87,406 | 9.8 | 20.2 | 9.0 | 61.0 |  |
| Toothbrushing after lunch | Yes | 103,771 | 15.2 | 22.7 | 10.9 | 51.1 | <0.001 |
|  | No | 65,883 | 10.4 | 20.1 | 10.5 | 59.0 |  |
| Toothbrushing after dinner/before bedtime | Yes | 160,236 | 13.8 | 22.1 | 10.8 | 53.3 | <0.001 |
|  | No | 9,418 | 7.2 | 15.8 | 9.0 | 67.9 |  |
| Chewing difficulty | Present | 37,704 | 1.0 | 17.2 | 1.6 | 80.3 | <0.001 |
|  | Absent | 131,950 | 16.3 | 22.8 | 12.8 | 48.0 |  |
| **Community-level contextual factors** | | | |  |  |  |  |
| Residential area | Dong | 86,771 | 14.1 | 22.1 | 10.8 | 53.0 | <0.001 |
|  | Eup/Myeon | 82,883 | 11.3 | 20.8 | 10.6 | 57.3 |  |
| Urban-rural classification | Special metropolitan city (Gu) | 14,761 | 16.1 | 22.5 | 11.5 | 49.9 | <0.001 |
|  | Metropolitan city (Gu) | 60,480 | 13.5 | 22.2 | 10.6 | 53.7 |  |
|  | City with <300,000 population | 6,246 | 13.7 | 20.7 | 10.8 | 54.8 |  |
|  | Urban-rural mixed city | 27,394 | 11.8 | 20.8 | 10.7 | 56.7 |  |
|  | County | 49,231 | 11.5 | 20.3 | 10.8 | 57.4 |  |
|  | County with a public medical institution | 11,542 | 10.6 | 19.5 | 10.6 | 59.3 |  |
| Integrated care pilot area | Yes | 69,289 | 12.6 | 21.2 | 10.6 | 55.5 | <0.001 |
|  | No | 100,365 | 13.9 | 22.1 | 10.9 | 53.2 |  |
| Medically underserved area | Yes | 73,713 | 10.9 | 19.7 | 10.9 | 58.4 | <0.001 |
|  | No | 95,941 | 13.9 | 22.2 | 10.7 | 53.2 |  |
| Depopulation area | Not designated | 90,982 | 14.0 | 22.2 | 10.8 | 52.9 | <0.001 |
|  | At-risk or declining area | 67,330 | 10.9 | 19.4 | 10.4 | 59.3 |  |
|  | Area of concern | 11,342 | 12.1 | 20.3 | 10.4 | 57.1 |  |
|  | |  |  |  |  |  |  |

Notes: n values are unweighted counts; percentages are survey-weighted. In Sensitivity Analysis A, fair, poor, and very poor responses were classified as poor/unhealthy, whereas good and very good responses were classified as good/healthy.

| **Supplementary Table 4. Sensitivity Analysis A: survey-weighted multinomial logistic regression when fair responses were classified as poor/unhealthy** | | | | | |
| --- | --- | --- | --- | --- | --- |
| Variable | Category (reference) | Poor oral only vs good both, AOR (95% CI) | Poor general only vs good both, AOR (95% CI) | Poor both vs good both, AOR (95% CI) | p-value |
| Sociodemographic factors |  |  |  |  |  |
| Sex | Female | 0.9 (0.8–0.9) | 1.2 (1.2–1.3) | 1.1 (1.0–1.1) | <0.001 |
| Age (years) | 45–54 | 1.0 (1.0–1.1) | 0.9 (0.9–1.0) | 0.9 (0.9–1.0) |  |
|  | 55–64 | 0.9 (0.9–1.0) | 0.8 (0.8–0.9) | 0.8 (0.7–0.8) | <0.001 |
|  | 65–74 | 1.0 (0.9–1.1) | 0.7 (0.6–0.8) | 0.8 (0.7–0.8) |  |
| Education level | ≤Elementary school | 1.6 (1.4–1.7) | 1.8 (1.6–2.0) | 2.7 (2.4–2.9) | <0.001 |
|  | Middle school | 1.3 (1.2–1.5) | 1.5 (1.4–1.7) | 2.0 (1.8–2.1) |  |
|  | High school | 1.2 (1.1–1.2) | 1.2 (1.2–1.3) | 1.5 (1.4–1.5) |  |
| Household income | <1M | 1.2 (1.1–1.4) | 1.4 (1.2–1.6) | 1.9 (1.7–2.1) | <0.001 |
|  | 1–<2M | 1.2 (1.1–1.3) | 1.3 (1.2–1.4) | 1.5 (1.4–1.6) |  |
|  | 2–<3M | 1.2 (1.1–1.3) | 1.2 (1.1–1.3) | 1.3 (1.2–1.4) |  |
|  | 3–<4M | 1.1 (1.1–1.2) | 1.1 (1.0–1.2) | 1.2 (1.1–1.2) |  |
| Spouse | Without | 1.0 (0.9–1.0) | 1.0 (0.9–1.1) | 1.0 (1.0–1.1) | 0.091 |
| Economic activity | No | 1.1 (1.0–1.1) | 1.3 (1.3–1.4) | 1.4 (1.3–1.5) | <0.001 |
| Health behaviors |  |  |  |  |  |
| Smoking | Yes | 1.4 (1.3–1.5) | 1.2 (1.1–1.3) | 1.5 (1.4–1.6) | <0.001 |
| Drinking | Yes | 1.1 (1.0–1.1) | 0.9 (0.8–0.9) | 0.9 (0.8–0.9) | <0.001 |
| Health status and need factors | |  |  |  |  |
| Mobility limitation | Yes | 1.5 (1.3–1.7) | 4.5 (4.0–5.0) | 5.2 (4.7–5.8) | <0.001 |
| History of hypertension diagnosis | Yes | 0.9 (0.9–1.0) | 1.6 (1.5–1.7) | 1.4 (1.4–1.5) | <0.001 |
| History of diabetes diagnosis | Yes | 1.1 (1.0–1.2) | 2.3 (2.1–2.5) | 2.4 (2.3–2.6) | <0.001 |
| Depression | Yes | 1.0 (0.9–1.2) | 1.8 (1.6–2.0) | 1.8 (1.6–2.1) | <0.001 |
| Stress | Yes | 1.0 (1.0–1.1) | 1.9 (1.7–2.0) | 2.0 (1.9–2.1) | <0.001 |
| Unmet medical needs | Yes | 1.2 (1.0–1.4) | 1.5 (1.3–1.8) | 1.7 (1.5–2.0) | <0.001 |
| Oral healthcare access and oral function | | |  |  |  |
| Unmet dental needs | Yes | 3.5 (3.1–3.9) | 1.1 (0.9–1.2) | 3.8 (3.4–4.2) | <0.001 |
| Dental scaling in the past year | No | 1.2 (1.1–1.2) | 1.0 (0.9–1.0) | 1.3 (1.2–1.3) | <0.001 |
| Toothbrushing after lunch | No | 1.1 (1.1–1.2) | 1.2 (1.1–1.3) | 1.3 (1.2–1.3) | <0.001 |
| Toothbrushing after dinner/before bedtime | No | 1.0 (0.9–1.2) | 1.1 (0.9–1.3) | 1.2 (1.1–1.4) | <0.001 |
| Community-level contextual factors | |  |  |  |  |
| Residential area | Eup/Myeon | 0.9 (0.9–1.0) | 1.0 (0.9–1.1) | 1.0 (1.0–1.1) | <0.05 |
| Urban-rural classification | Metropolitan city (Gu) | 1.1 (1.1–1.2) | 1.1 (1.0–1.2) | 1.3 (1.2–1.4) | <0.001 |
|  | City with <300,000 population | 1.0 (0.9–1.1) | 1.1 (0.9–1.2) | 1.2 (1.0–1.3) |  |
|  | Urban-rural mixed city | 1.1 (1.0–1.2) | 1.2 (1.1–1.3) | 1.3 (1.2–1.4) |  |
|  | County | 1.1 (0.9–1.2) | 1.1 (1.0–1.3) | 1.2 (1.1–1.4) |  |
|  | County with a public medical institution | 1.0 (0.9–1.2) | 1.1 (0.9–1.3) | 1.1 (1.0–1.3) |  |
| Integrated care pilot area | No | 0.9 (0.9–0.9) | 0.9 (0.9–1.0) | 0.9 (0.8–0.9) | <0.001 |
| Medically underserved area | Yes | 1.0 (0.9–1.1) | 1.1 (1.0–1.2) | 1.0 (0.9–1.1) | <0.01 |
| Depopulation area | At-risk or declining area | 1.0 (0.9–1.0) | 0.9 (0.8–1.0) | 1.0 (0.9–1.0) | 0.166 |
|  | Area of concern | 1.0 (0.9–1.1) | 0.9 (0.9–1.0) | 1.0 (0.9–1.1) |  |

Notes: The analysis used the full complete-case sample. In Sensitivity Analysis A, fair responses were classified as poor/unhealthy. Chewing difficulty was not included as a predictor.

AOR = adjusted odds ratio; CI = confidence interval. KCHS sampling weights, strata, and primary sampling units were applied.

| **Supplementary Table 5. Sensitivity Analysis B: survey-weighted distribution when fair responses were classified as good/healthy** | | | | | | | |
| --- | --- | --- | --- | --- | --- | --- | --- |
| Characteristic | Category | n (unweighted) | Good both (weighted %) | Poor oral only (weighted %) | Poor general only (weighted %) | Poor both (weighted %) | p-value |
| Total |  | 169,654 | 60.2 | 20.0 | 8.7 | 11.1 |  |
| **Sociodemographic factors** | |  |  |  |  |  |  |
| Sex | Male | 76,017 | 59.8 | 23.4 | 6.8 | 10.0 | <0.001 |
|  | Female | 93,637 | 60.6 | 16.8 | 10.5 | 12.1 |  |
| Age (years) | 45–54 | 36,962 | 73.5 | 17.1 | 5.5 | 3.9 | <0.001 |
|  | 55–64 | 49,355 | 65.0 | 20.9 | 7.0 | 7.2 |  |
|  | 65–74 | 45,475 | 53.6 | 22.0 | 11.1 | 13.3 |  |
|  | ≥75 years | 37,862 | 34.3 | 20.9 | 15.1 | 29.7 |  |
| Education level | ≤Elementary school | 44,822 | 32.7 | 21.8 | 15.7 | 29.7 | <0.001 |
|  | Middle school | 24,865 | 49.5 | 23.2 | 11.4 | 15.9 |  |
|  | High school | 55,174 | 63.1 | 20.9 | 7.9 | 8.0 |  |
|  | ≥College | 44,793 | 73.4 | 17.2 | 5.4 | 4.1 |  |
| Household income  (million KRW) | <1M | 25,333 | 28.9 | 20.2 | 16.6 | 34.3 | <0.001 |
|  | 1–<2M | 29,637 | 42.8 | 22.3 | 13.7 | 21.3 |  |
|  | 2–<3M | 25,725 | 55.8 | 21.9 | 10.3 | 12.1 |  |
|  | 3–<4M | 21,373 | 62.4 | 20.8 | 8.4 | 8.4 |  |
|  | ≥4M | 67,586 | 71.0 | 18.7 | 5.7 | 4.7 |  |
| Spouse | With | 122,713 | 64.4 | 19.7 | 7.7 | 8.2 | <0.001 |
|  | Without | 46,941 | 47.5 | 20.8 | 11.8 | 19.9 |  |
| Economic activity | Yes | 102,953 | 67.6 | 21.2 | 5.6 | 5.6 | <0.001 |
|  | No | 66,701 | 48.6 | 18.2 | 13.5 | 19.7 |  |
| **Health behaviors** | | |  |  |  |  |  |
| Smoking | Yes | 25,771 | 53.4 | 29.0 | 6.3 | 11.3 | <0.001 |
|  | No | 143,883 | 61.6 | 18.2 | 9.2 | 11.1 |  |
| Drinking | Yes | 94,635 | 66.5 | 21.0 | 5.9 | 6.6 | <0.001 |
|  | No | 75,019 | 49.7 | 18.4 | 13.4 | 18.5 |  |
| **Health status and need factors** | | |  |  |  |  |  |
| Mobility limitation | Yes | 34,881 | 22.3 | 15.6 | 22.3 | 39.8 | <0.001 |
|  | No | 134,773 | 66.9 | 20.8 | 6.3 | 6.0 |  |
| History of hypertension diagnosis | Yes | 71,119 | 50.7 | 19.9 | 12.4 | 17.1 | <0.001 |
|  | No | 98,535 | 65.8 | 20.1 | 6.6 | 7.6 |  |
| History of diabetes diagnosis | Yes | 31,333 | 41.7 | 19.9 | 15.5 | 22.8 | <0.001 |
|  | No | 138,321 | 63.8 | 20.0 | 7.4 | 8.8 |  |
| Depression | Yes | 12,195 | 36.3 | 17.6 | 17.9 | 28.3 | <0.001 |
|  | No | 157,459 | 62.1 | 20.2 | 8.0 | 9.8 |  |
| Stress | Yes | 30,562 | 47.8 | 19.5 | 13.2 | 19.5 | <0.001 |
|  | No | 139,092 | 63.2 | 20.1 | 7.6 | 9.1 |  |
| Unmet medical needs | Yes | 7,606 | 42.9 | 22.9 | 11.3 | 22.9 | <0.001 |
|  | No | 162,048 | 61.0 | 19.8 | 8.6 | 10.6 |  |
| **Oral healthcare access and oral function** | | |  |  |  |  |  |
| Unmet dental needs | No | 148,185 | 63.5 | 18.0 | 9.0 | 9.6 | <0.001 |
|  | Yes | 21,469 | 36.9 | 34.4 | 6.9 | 21.7 |  |
| Dental scaling in the past year | Yes | 82,248 | 68.0 | 17.4 | 8.1 | 6.5 | <0.001 |
|  | No | 87,406 | 50.5 | 23.2 | 9.4 | 16.9 |  |
| Toothbrushing after lunch | Yes | 103,771 | 63.9 | 18.8 | 8.2 | 9.2 | <0.001 |
|  | No | 65,883 | 53.5 | 22.2 | 9.6 | 14.7 |  |
| Toothbrushing after dinner/before bedtime | Yes | 160,236 | 61.2 | 19.7 | 8.7 | 10.4 | <0.001 |
|  | No | 9,418 | 38.4 | 25.0 | 9.9 | 26.6 |  |
| Chewing difficulty | Present | 37,704 | 14.6 | 42.1 | 5.2 | 38.0 | <0.001 |
|  | Absent | 131,950 | 70.5 | 15.0 | 9.5 | 5.0 |  |
| **Community-level contextual factors** | | |  |  |  |  |  |
| Residential area | Dong | 86,771 | 62.0 | 19.5 | 8.4 | 10.2 | <0.001 |
|  | Eup/Myeon | 82,883 | 53.8 | 21.8 | 9.9 | 14.6 |  |
| Urban-rural classification | Special metropolitan city (Gu) | 14,761 | 65.3 | 17.5 | 8.4 | 8.8 | <0.001 |
|  | Metropolitan city (Gu) | 60,480 | 61.0 | 20.0 | 8.4 | 10.5 |  |
|  | City with <300,000 population | 6,246 | 61.3 | 20.2 | 8.2 | 10.3 |  |
|  | Urban-rural mixed city | 27,394 | 53.8 | 21.5 | 10.2 | 14.5 |  |
|  | County | 49,231 | 53.8 | 21.5 | 10.2 | 14.5 |  |
|  | County with a public medical institution | 11,542 | 49.4 | 22.4 | 11.1 | 17.1 |  |
| Integrated care pilot area | Yes | 69,289 | 59.1 | 20.4 | 8.8 | 11.7 | <0.001 |
|  | No | 100,365 | 60.7 | 19.8 | 8.7 | 10.8 |  |
| Medically underserved area | Yes | 73,713 | — | — | — | — | <0.001 |
|  | No | 95,941 | 61.4 | 19.7 | 8.5 | 10.4 |  |
| Depopulation area | Not designated | 90,982 | 61.7 | 19.7 | 8.4 | 10.2 | <0.001 |
|  | At-risk or declining area | 67,330 | 51.2 | 22.0 | 10.4 | 16.3 |  |
|  | Area of concern | 11,342 | 56.6 | 20.2 | 9.3 | 13.9 |  |
|  | | |  |  |  |  |  |

Notes: n values are unweighted counts; percentages are survey-weighted. In Sensitivity Analysis B, very good, good, and fair responses were classified as good/healthy, whereas poor and very poor responses were classified as poor/unhealthy.

| **Supplementary Table 6. Sensitivity Analysis B: survey-weighted multinomial logistic regression when fair responses were classified as good/healthy** | | | | |  |  |  |  |
| --- | --- | --- | --- | --- | --- | --- | --- | --- |
| Variable | Category (reference) | Poor oral only vs good both | Poor general only vs good both | Poor both vs good both | p-value |  |  |  |
| Sociodemographic factors |  |  |  |  |  |  |  |  |
| Sex | Female | 0.7 (0.7–0.8) | 1.0 (0.9–1.0) | 0.7 (0.6–0.7) | <0.001 |  |  |  |
| Age (years) | 55–64 | 1.3 (1.3–1.4) | 1.0 (0.9–1.0) | 1.3 (1.2–1.4) |  |  |  |  |
|  | 65–74 | 1.4 (1.4–1.5) | 1.4 (1.4–1.5) | 0.9 (0.8–1.0) | <0.001 |  |  |  |
|  | ≥75 | 1.7 (1.6–1.8) | 0.9 (0.8–0.9) | 1.7 (1.5–1.8) |  |  |  |  |
| Education level | ≤Elementary school | 1.9 (1.7–2.0) | 1.9 (1.8–2.1) | 3.1 (2.8–3.4) | <0.001 |  |  |  |
|  | Middle school | 1.5 (1.4–1.6) | 1.5 (1.4–1.6) | 2.1 (1.9–2.3) |  |  |  |  |
|  | High school | 1.2 (1.2–1.3) | 1.3 (1.2–1.4) | 1.5 (1.4–1.6) |  |  |  |  |
| Household income  (million KRW) | <1M | 1.4 (1.3–1.5) | 1.8 (1.6–2.0) | 2.2 (2.1–2.4) | <0.001 |  |  |  |
|  | 1–<2M | 1.2 (1.1–1.3) | 1.5 (1.4–1.6) | 1.7 (1.6–1.9) |  |  |  |  |
|  | 2–<3M | 1.1 (1.0–1.2) | 1.3 (1.2–1.4) | 1.4 (1.3–1.5) |  |  |  |  |
|  | 3–<4M | 1.0 (1.0–1.1) | 1.2 (1.1–1.3) | 1.2 (1.1–1.3) |  |  |  |  |
| Spouse | Without | 1.1 (1.0–1.1) | 1.1 (1.0–1.1) | 1.3 (1.2–1.3) | <0.001 |  |  |  |
| Economic activity | No | 1.0 (1.0–1.1) | 1.9 (1.8–2.0) | 2.0 (1.9–2.2) | <0.001 |  |  |  |
| Health behaviors |  |  |  |  |  |  |  |  |
| Smoking | Yes | 1.6 (1.5–1.7) | 1.1 (1.0–1.2) | 1.5 (1.4–1.6) | <0.001 |  |  |  |
| Drinking | Yes | 1.0 (0.9–1.0) | 0.5 (0.5–0.6) | 0.6 (0.5–0.6) | <0.001 |  |  |  |
| Health status and need factors |  |  |  |  |  |  |  |  |
| Mobility limitation | Yes | 1.5 (1.4–1.6) | 4.9 (4.7–5.3) | 6.1 (5.8–6.5) | <0.001 |  |  |  |
| History of hypertension diagnosis | Yes | 1.0 (1.0–1.0) | 1.5 (1.4–1.6) | 1.4 (1.3–1.5) | <0.001 |  |  |  |
| History of diabetes diagnosis | Yes | 1.3 (1.2–1.3) | 2.3 (2.2–2.5) | 2.6 (2.4–2.7) | <0.001 |  |  |  |
| Depression | Yes | 1.2 (1.1–1.3) | 2.2 (2.0–2.3) | 2.2 (2.1–2.4) | <0.001 |  |  |  |
| Stress | Yes | 1.3 (1.2–1.4) | 2.5 (2.3–2.6) | 3.1 (2.9–3.3) | <0.001 |  |  |  |
| Unmet medical needs | Yes | 1.1 (1.0–1.2) | 1.6 (1.4–1.8) | 1.7 (1.5–1.9) | <0.001 |  |  |  |
|  | | | |  |  |  |  |  |
| Unmet dental needs | Yes | 3.0 (2.9–3.2) | 1.1 (1.0–1.2) | 2.9 (2.7–3.2) | <0.001 |  |  |  |
| Dental scaling in the past year | No | 1.3 (1.2–1.3) | 1.0 (1.0–1.1) | 1.5 (1.4–1.6) | <0.001 |  |  |  |
| Toothbrushing after lunch | No | 1.1 (1.0–1.1) | 1.1 (1.0–1.2) | 1.2 (1.1–1.2) | <0.001 |  |  |  |
| Toothbrushing after dinner/before bedtime | No | 1.3 (1.2–1.4) | 1.1 (1.0–1.2) | 1.5 (1.4–1.7) | <0.001 |  |  |  |
| Community-level contextual factors | | |  |  |  |  |  |  |
| Residential area | Eup/Myeon | 1.0 (0.9–1.0) | 1.0 (0.9–1.1) | 1.0 (0.9–1.1) | 0.973 |  |  |  |
| Urban-rural classification | Metropolitan city (Gu) | 1.2 (1.1–1.3) | 1.2 (1.1–1.3) | 1.4 (1.3–1.5) | <0.001 |  |  |  |
|  | City with <300,000 population | 1.2 (1.1–1.3) | 1.1 (1.0–1.3) | 1.3 (1.1–1.5) |  |  |  |  |
|  | Urban-rural mixed city | 1.3 (1.2–1.4) | 1.2 (1.1–1.4) | 1.5 (1.4–1.7) |  |  |  |  |
|  | County | 1.2 (1.1–1.3) | 1.2 (1.1–1.4) | 1.3 (1.2–1.5) |  |  |  |  |
|  | County with a public medical institution | 1.2 (1.0–1.3) | 1.3 (1.1–1.5) | 1.4 (1.2–1.6) |  |  |  |  |
| Integrated care pilot area | No | 1.0 (0.9–1.0) | 1.0 (1.0–1.1) | 1.0 (1.0–1.1) | 0.767 |  |  |  |
| Medically underserved area | Yes | 1.0 (0.9–1.0) | 0.9 (0.9–1.0) | 0.9 (0.8–1.0) | <0.05 |  |  |  |
| Depopulation area | At-risk or declining area | 1.0 (1.0–1.1) | 1.0 (1.0–1.1) | 1.1 (1.1–1.2) | <0.01 |  |  |  |
|  | Area of concern | 0.9 (0.9–1.0) | 1.0 (0.9–1.1) | 1.0 (0.9–1.1) |  |  |  |  |

Notes: The analysis used the full complete-case sample. In Sensitivity Analysis B, fair responses were classified as good/healthy. Chewing difficulty was not included as a predictor.

AOR = adjusted odds ratio; CI = confidence interval. KCHS sampling weights, strata, and primary sampling units were applied.

**Supplementary Table 7. Hidden-vulnerability outcomes across primary and sensitivity samples**

| **Sample** | **Reclassification group** | **Mobility limitation (%)** | **p-value** | **Unmet medical needs (%)** | **p-value** | **Chewing difficulty (%)** | **p-value** |
| --- | --- | --- | --- | --- | --- | --- | --- |
| Primary high-contrast sample | Both good | 2.4 | <0.001 | 2.0 | <0.001 | 1.3 | <0.001 |
| Primary high-contrast sample | Missed by SRH (good general / poor oral) | 7.3 |  | 3.7 |  | 34.8 |  |
| Primary high-contrast sample | Both poor | 54.0 |  | 9.0 |  | 5.0 |  |
| Sensitivity A: fair = poor/unhealthy | Both good | 2.4 | <0.001 | 2.0 | <0.001 | 1.2 | <0.001 |
| Sensitivity A: fair = poor/unhealthy | Missed by SRH (good general / poor oral) | 4.3 |  | 3.1 |  | 14.5 |  |
| Sensitivity A: fair = poor/unhealthy | Both poor | 22.5 |  | 5.6 |  | 27.4 |  |
| Sensitivity B: fair = good/healthy | Both good | 5.6 | <0.001 | 3.1 |  | 4.5 | <0.001 |
| Sensitivity B: fair = good/healthy | Missed by SRH (good general / poor oral) | 11.8 |  | 5.0 |  | 38.8 |  |
| Sensitivity B: fair = good/healthy | Both poor | 38.5 |  | 9.0 |  | 63.1 |  |

Notes: Values are survey-weighted percentages.

**Supplementary Table 8. Survey-weighted multinomial logistic regression results for the base model (Model 4; chewing difficulty excluded)**

| **Variable** | **Category (reference)** | **Poor oral only vs good both, AOR (95% CI)** | **Poor general only vs good both, AOR (95% CI)** | **Poor both vs good both, AOR (95% CI)** | **p-value** |
| --- | --- | --- | --- | --- | --- |
| **Sociodemographic factors** |  |  |  |  |  |
| Sex | Female | 0.7 (0.6–0.7) | 1.1 (1.0–1.2) | 0.8 (0.7–0.9) | <0.001 |
| Age (years) | 55–64 | 1.3 (1.2–1.4) | 0.9 (0.8–1.0) | 1.2 (1.1–1.4) |  |
|  | 65–74 | 1.3 (1.2–1.5) | 0.8 (0.7–0.9) | 1.1 (1.0–1.3) | <0.001 |
|  | ≥75 | 1.7 (1.5–1.9) | 0.7 (0.6–0.9) | 1.2 (1.1–1.4) |  |
| Education level | ≤Elementary school | 2.3 (2.1–2.6) | 2.8 (2.4–3.3) | 4.7 (4.2–5.4) | <0.001 |
|  | Middle school | 1.8 (1.6–2.0) | 2.0 (1.7–2.3) | 2.9 (2.6–3.3) |  |
|  | High school | 1.3 (1.2–1.5) | 1.5 (1.3–1.7) | 1.8 (1.6–2.0) |  |
| Household income (million KRW) | <1M | 1.5 (1.3–1.7) | 2.1 (1.7–2.5) | 3.0 (2.6–3.5) | <0.001 |
|  | 1–<2M | 1.3 (1.2–1.4) | 1.8 (1.5–2.0) | 2.2 (1.9–2.4) |  |
|  | 2–<3M | 1.2 (1.1–1.4) | 1.5 (1.3–1.8) | 1.7 (1.5–1.9) |  |
|  | 3–<4M | 1.1 (1.0–1.3) | 1.3 (1.2–1.6) | 1.4 (1.2–1.5) |  |
| Spouse | Without | 1.0 (0.9–1.1) | 1.1 (1.0–1.2) | 1.2 (1.1–1.3) | <0.01 |
| Economic activity | No | 1.0 (0.9–1.1) | 2.0 (1.8–2.2) | 2.2 (2.0–2.4) | <0.001 |
| **Health behaviors** |  |  |  |  |  |
| Smoking | Yes | 1.8 (1.7–2.0) | 1.3 (1.1–1.5) | 1.8 (1.6–2.0) | <0.001 |
| Drinking | Yes | 1.0 (0.9–1.0) | 0.5 (0.5–0.6) | 0.6 (0.5–0.6) | <0.001 |
| **Health status and need factors** |  |  |  |  |  |
| Mobility limitation | Yes | 2.0 (1.8–2.3) | 10.6 (9.2–12.2) | 12.6 (11.2–14.2) | <0.001 |
| History of hypertension diagnosis | Yes | 0.9 (0.8–1.0) | 1.8 (1.6–1.9) | 1.6 (1.5–1.7) | <0.001 |
| History of diabetes diagnosis | Yes | 1.3 (1.2–1.5) | 3.6 (3.2–4.1) | 4.1 (3.7–4.5) | <0.001 |
| Depression | Yes | 1.2 (1.1–1.5) | 2.9 (2.4–3.4) | 3.2 (2.8–3.8) | <0.001 |
| Stress | Yes | 1.3 (1.1–1.4) | 3.4 (3.0–3.8) | 4.0 (3.6–4.4) | <0.001 |
| Unmet medical needs | Yes | 1.1 (0.9–1.4) | 2.0 (1.6–2.5) | 2.2 (1.8–2.7) | <0.001 |
| Oral healthcare access and oral function |  |  |  |  |  |
| Unmet dental needs | Yes | 6.0 (5.3–6.7) | 1.4 (1.1–1.7) | 6.2 (5.4–7.0) | <0.001 |
| Dental scaling in the past year | No | 1.3 (1.3–1.4) | 0.9 (0.8–1.0) | 1.5 (1.4–1.6) | <0.001 |
| Toothbrushing after lunch | No | 1.2 (1.1–1.3) | 1.2 (1.1–1.3) | 1.3 (1.2–1.3) | <0.001 |
| Toothbrushing after dinner/before bedtime | No | 1.2 (1.0–1.4) | 1.3 (1.0–1.6) | 1.6 (1.3–1.9) | <0.001 |

Notes: Model 4 includes sociodemographic, behavioral, health status and need, and oral healthcare access variables. Chewing difficulty is excluded. Community-level contextual variables are not included in Model 4.

Reference categories match Table 2.

**Supplementary Table 9. Variance inflation factor (VIF) diagnostics for the final regression model**

| **Variable** | **VIF** |
| --- | --- |
| **Sex** | 1.386 |
| **Age** | 2.259 |
| **Education level** | 2.144 |
| **Household income** | 1.867 |
| **Spouse** | 1.282 |
| **Economic activity** | 1.360 |
| **Smoking** | 1.253 |
| **Drinking** | 1.275 |
| **Mobility limitation** | 1.582 |
| **Hypertension** | 1.192 |
| **Diabetes** | 1.092 |
| **Depression** | 1.145 |
| **Stress** | 1.143 |
| **Unmet medical needs** | 1.090 |
| **Unmet dental needs** | 1.127 |
| **Dental scaling in past year** | 1.241 |
| **Toothbrushing after lunch** | 1.111 |
| **Toothbrushing after dinner/before bedtime** | 1.094 |
| **Residential area** | 2.777 |
| **Urban-rural classification** | 4.011 |
| **Integrated care pilot area** | 1.112 |
| **Medically underserved area** | 2.959 |
| **Depopulation area** | 1.498 |

Notes: VIF values ranged from 1.090 to 4.011.
